# EHMTI-0237. The A11 hypothalamic nucleus is susceptible to nitric oxide signalling

**DOI:** 10.1186/1129-2377-15-S1-F2

**Published:** 2014-09-18

**Authors:** AP Andreou, JH Chamberlain, JV Torres-Perez, F Noormohamed, PJ Goadsby, C Bantel, I Nagy

**Affiliations:** 1 Headache Research- Section of Anaesthetics Pain Medicine and Intensive Care Chelsea and Westminster Hospital Faculty of Medicine, Imperial College London, London, UK; 2 Headache Group NIHR-Wellcome Trust Clinical Research Facility, King’s College London, London, UK

## Introduction

The involvement of hypothalamic nuclei in the pathophysiology of migraine and trigeminal autonomic cephalalgias has been suggested for some time, with dopaminergic mechanisms proposed to play a prominent role. The hypothalamic A11 nucleus is, to date, the only nucleus found to provide a dopamine-mediated inhibitory effect on nociceptive transmission in the trigeminocervical complex.

## Aims

We aimed to examine the effects of trigeminovascular stimulation and nitrergic mechanisms on the neuronal activity of A11 hypothalamic neurons.

## Methods

In anesthetised male Sprague-Dawley rats, extracellular single neuron electrophysiological recordings were performed from the A11 hypothalamic area. Trigeminovascular activation was achieved by electrical stimulation of the superior sagittal sinus. The nitric oxide donor sodium nitroprusside (SNP), or saline were intravenously infused and their effects on spontaneous neuronal firing examined. In a different experimental group, animals were pre-treated with indomethacin 15 minutes prior SNP infusion.

## Results

Infusion of SNP significantly attenuated spontaneous neuronal firing in the A11 nucleus for at least 90 minutes, whereas saline infusion had no significant effects. Pre-treatment with indomethacin, significantly blocked the SNP-induced attenuation of spontaneous neuronal activity. None of the recorded neurons, located in the rostrocaudal extent of the A11 nucleus, demonstrated increased evoked-activity in response to electrical activation of the superior sagittal sinus.

## Conclusions

The A11 hypothalamic nucleus is susceptible to nitric oxide-mediated inhibition and at least, part of indomethacin’s mechanism of action involves interactions with nitric oxide signaling. Our data further suggest that the A11 nucleus may not be activated directly by trigeminohypothalamic afferents.

No conflict of interest.

